# Peer Review of “The Association of Shared Care Networks With 30-Day Heart Failure Excessive Hospital Readmissions: Longitudinal Observational Study”

**DOI:** 10.2196/37003

**Published:** 2022-04-06

**Authors:** Mahin Nomali

**Affiliations:** 1 Department of Epidemiology and Biostatistics School of Public Health Tehran University of Medical Sciences Tehran Iran

**Keywords:** patient readmission, quality assurance, health care, catchment area, health, community networks, regional medical programs

*This is a peer-review report submitted for the paper “The Association of Shared Care Networks With 30-Day Heart Failure Excessive Hospital Readmissions: Longitudinal Observational Study.”*

## Round 1 Review

### Major Comments

- Title: For this study [1], please include the type of study in the title. If you are considering 30-day readmission, please specify it in the title.

### Abstract

- Please move the objective section to the end of the background section, and it is recommended that it is written the same as in the study title.
- Methods: Please start this section with the study design. Study setting, study variables, and outcomes and their measurements should be mentioned, briefly. Eligibility criteria have not been provided.
- Methods: Excessive readmission ratio: I think it is excessive readmission risk ratio because no person-year has been reported. Thus, to improve the reporting, please revise it in the whole document.
- Results: To facilitate the interpretation of the study results, please convert beta coefficients by exponentiating them.
- Please use expanded forms of the abbreviations the first time they are mentioned. The expanded form of some abbreviations has not been provided.
- Keywords: Please write these according to the Medical Subject Headings (MeSH) system.
- Introduction: The necessity of this study is not clear. Please provide a paragraph about the importance and necessity of this study and why you designed and conducted this study.
- Methods: It is recommended to write this section according to the STROBE (Strengthening the Reporting of Observational Studies in Epidemiology) standard writing and refer to it in the first paragraph of the Methods section.
- Please start this section with the study design. A retrospective study is not a study design and refers to the type of data collection.
- Please provide information about institutional review board (IRB) approval of this study.
- Study variables and their measurement should be provided.
- Statistical analysis: please use converted forms of beta coefficients.
- Results: The Results section is very long. Please avoid providing data both in the text and the table.
- Please use converted forms of beta coefficients in the Results section.
- Please identify adjusted and unadjusted beta coefficients in the Results section both in the Abstract and full text.
- I do not think there is a “perspective section” in the JMIR structure. You can add it to the Discussion and Conclusion section if it is necessary.
- Tables: They are not in the scientific form. Please revise them according to JMIR guidelines.

## Round 2 Review

I would like to thank the authors for considering all the reviewers’ comments.

However, there is no IRB or research ethics committee approval.

According to the authors' statement “all data used in this work is made publicly available by the Hospital Reduction Readmission Program (HRRP) and Office of Statewide Health Planning and Development (OSHPD).” It is recommended to mention it in the Acknowledgments section and the first paragraph of the study design.

## Abbreviations

HRRP: Hospital Reduction Readmission Program
IRB: institutional review board
MeSH: Medical Subject Headings
OSHPD: Office of Statewide Health Planning and Development
STROBE: Strengthening the Reporting of Observational Studies in Epidemiology
